# Successful awake video-laryngoscopic intubation in a patient with a giant mobile vocal process granuloma causing dynamic glottic obstruction: A case report

**DOI:** 10.1097/MD.0000000000046656

**Published:** 2025-12-12

**Authors:** Jinyoung Oh, Sou-Hyun Lee, Kyung-Hwa Kwak, Younghoon Jeon, Jongone Park, Sung-Hye Byun

**Affiliations:** a Department of Anesthesiology and Pain Medicine, School of Medicine, Kyungpook National University, Kyungpook National University Chilgok Hospital, Daegu, Republic of Korea; b Department of Anesthesiology and Pain Medicine, School of Medicine, Kyungpook National University, Kyungpook National University Hospital, Daegu, Republic of Korea.

**Keywords:** airway management, awake intubation, difficult airway, video laryngoscope, vocal process granuloma

## Abstract

**Rationale:**

Vocal process granulomas are benign laryngeal lesions that are often asymptomatic but can cause critical airway obstruction upon rapid enlargement, although rare. In such cases, conventional induction of general anesthesia may lead to complete airway collapse. Therefore, careful selection of the anesthesia induction technique used is crucial to ensure patient safety, with awake intubation typically being preferred.

**Patient concerns:**

A 64-year-old woman presented to the emergency department with progressive dyspnea and stridor over 3 days. She had a history of hoarseness following phacoemulsification under general anesthesia with endotracheal intubation.

**Diagnoses:**

Follow-up laryngoscopy revealed a giant, stalked granuloma attached to the posterior one-third of the left vocal cord, nearly obstructing the glottic opening. Preinduction fiberoptic bronchoscopy (FOB) confirmed near-complete dynamic glottic obstruction during inspiration.

**Interventions:**

Emergency laryngeal microscopic surgery was planned. Due to the high risk of airway compromise during induction, awake video laryngoscope (VL)-guided intubation was selected over FOB-guided intubation, as the latter was limited by its inability to provide simultaneous visualization of the lesion, glottis, and endotracheal tube, as well as by insufficient mechanical support. Airway preparation included topical lidocaine, superior laryngeal nerve block, and transtracheal block. Remifentanil infusion and high-flow nasal oxygen were used to optimize patient comfort and oxygenation. An endotracheal tube with an internal diameter of 5.5 mm was successfully advanced during exhalation using the VL-guided approach.

**Outcomes:**

The surgery was completed without complications. At the 2-week, 2-month, and 1-year follow-ups, the patient remained asymptomatic, and laryngoscopy showed a well-healed surgical site.

**Lessons:**

Awake VL-guided intubation can be a safe and effective alternative to FOB for managing critical airways caused by giant, mobile vocal process granulomas. This technique offers continuous visualization, facilitates safer tube advancement, and reduces the risk of trauma and highlights the importance of integrating multidisciplinary collaboration and meticulous preinduction planning to optimize airway management.

## 1. Introduction

Vocal process granulomas are benign laryngeal lesions commonly associated with trauma, such as prolonged intubation, voice strain, or gastroesophageal reflux.^[1,2]^ While typically asymptomatic or causing mild hoarseness, rapid enlargement may lead to critical airway compromise. In such cases, conventional induction of general anesthesia can result in complete airway obstruction due to dynamic lesion displacement and loss of spontaneous ventilation. Awake intubation techniques, particularly those utilizing video laryngoscope (VL), offer safer alternatives by preserving airway patency during the procedure.^[3]^ The VL offers a broad, shared view of the dynamic glottic anatomy, allowing precise timing and controlled advancement of the endotracheal tube (ETT) in patients with large, mobile lesions. Herein, we present a case of a giant, mobile vocal process granuloma causing near-complete glottic obstruction, which was successfully managed with awake VL-guided intubation followed by emergency laryngeal surgery.

## 2. Case presentation

**Figure 1.**
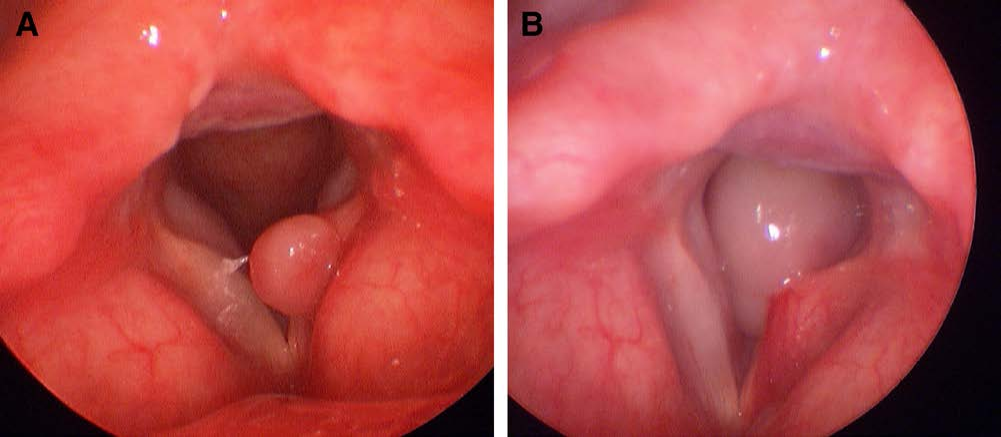
(A) Laryngoscopic image obtained at the otolaryngology clinic during evaluation for hoarseness 2 months after tracheal intubation showing a post-intubation granuloma on the vocal cord. (B) Follow-up laryngoscopic image at the time of presentation with progressive dyspnea, demonstrating a markedly enlarged, stalked granuloma attached to the posterior one-third of the left vocal cord, nearly obstructing the glottic opening.

A 64-year-old woman (148 cm, 57 kg) presented to the emergency department of our hospital with the chief complaint of progressive dyspnea for 3 days. Four months ago, the patient underwent tracheal intubation using a 7.0 mm internal diameter (ID) ETT under general anesthesia for phacoemulsification. Two months later, the patient presented to the otolaryngology clinic with hoarseness and was subsequently diagnosed with post-intubation granuloma (Fig. 1A). However, at that time, the hoarseness had partially improved, so conservative management was selected. The patient also had a history of panic disorder but was not taking any antidepressant medication and had no other significant medical or family history.

A follow-up laryngoscopy at the otolaryngology clinic revealed a granuloma attached to the posterior one-third of the left vocal cord by a stalk, which had enlarged to obstruct most of the glottic opening (Fig. 1B). Stridor and severe hoarseness were noted. The patient complained of dyspnea even in a sitting position, but peripheral oxygen saturation remained above 95%. The patient was slightly nervous, but able to maintain a supine position. Airway evaluation revealed an inter-incisor gap of 5 cm and Mallampati class IV. The patient also exhibited limited neck mobility; a short, thick neck; and fair dental status. Blood laboratory tests were within normal limits and chest radiography showed no abnormalities. Given the patient’s symptoms, emergency laryngeal microscopic surgery was planned. The attending anesthesiologists recommended a tracheostomy under local anesthesia followed by general anesthesia because of the high risk of airway failure during induction of general anesthesia. However, the otolaryngologist requested that tracheal intubation be attempted first due to concerns regarding tracheostomy-related complications. Thus, it was decided that the otolaryngology team would be on stand-by to perform an immediate tracheostomy if intubation failed to secure the airway.

The patient received no sedative premedication but was administered glycopyrrolate 0.1 mg to reduce airway secretions. Upon arrival in the operating room, standard monitoring was conducted, including noninvasive blood pressure, electrocardiogram, and peripheral oxygen saturation, while applying high-flow nasal oxygen. Due to patient anxiety, the blood pressure (185/98 mm Hg) and pulse rate (95 beats/min) were initially elevated, but gradually decreased to 151/72 mm Hg and 76 beats/min, respectively. A bispectral index sensor was placed on the patient’s forehead in advance for monitoring the depth of anesthesia during the postanesthetic induction period. Preoperative laryngoscope revealed insufficient space around the glottis for standard adult-sized ETT due to the enlarged mass. Therefore, a range of ETTs with IDs ranging from 5.0 to 6.0 mm were prepared.

**Figure 2.**
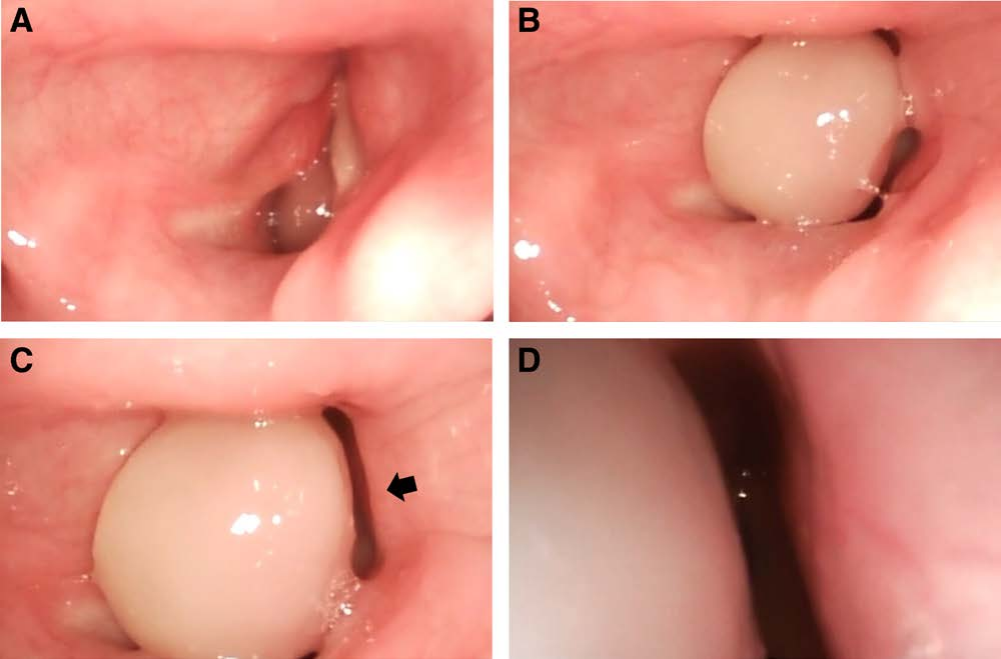
Preinduction fiberoptic bronchoscopic views of the vocal process granuloma, obtained by anesthesiologists during airway evaluation. (A) During inspiration, the granuloma prolapses into the glottic inlet, partially obstructing the airway. (B) During expiration, the granuloma retracts outward from the glottic inlet, relieving the obstruction. (C) As the bronchoscope advances closer, a narrow space is observed on the right side of the granuloma (indicated by arrow). (D) The bronchoscope is further advanced through the right-sided gap (corresponding to the arrow in C) to assess the feasibility of endotracheal tube passage without lesion dislodgment or trauma.

Since fiberoptic bronchoscopy (FOB) could not facilitate simultaneous visualization of the mobile granuloma’s movement and position, the glottis, and the ETT during advancement, and offered insufficient mechanical support for controlled navigation around the lesion during awake intubation, a VL-guided technique was selected. For airway preparation, topical anesthesia was applied to the oropharynx, including the posterior aspect of the tongue base, using 3 pumps of 10% lidocaine spray, and a bilateral superior laryngeal nerve block was performed under ultrasound guidance using 2 mL of 2% lidocaine. A translaryngeal block was also performed by penetrating the cricothyroid membrane and administering 4 mL of 2% lidocaine to block the recurrent laryngeal nerve. Although VL-guided awake intubation had been selected in advance, the anesthesiologist performed a preinduction FOB after airway preparation to assess the lesion’s dynamic behavior and the degree of glottic obstruction during respiration. This revealed that the mass dynamically prolapsed into the glottis during inspiration, causing near-complete obstruction (Fig. 2A–D).

**Figure 3.**
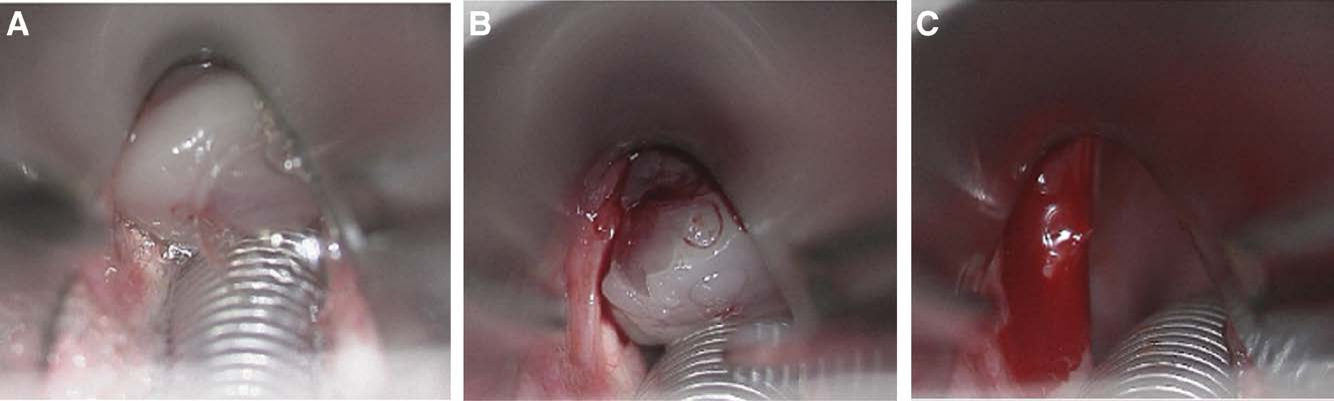
Intraoperative laryngoscopic findings. (A) Following awake intubation and subsequent induction of general anesthesia, a suspension laryngoscope was placed to expose the granuloma within the surgical field. (B) The pedunculated granuloma is visualized in full view prior to excision. (C) Post-excision image showing the complete removal of the granuloma with a clear glottic inlet.

**Figure 4.**
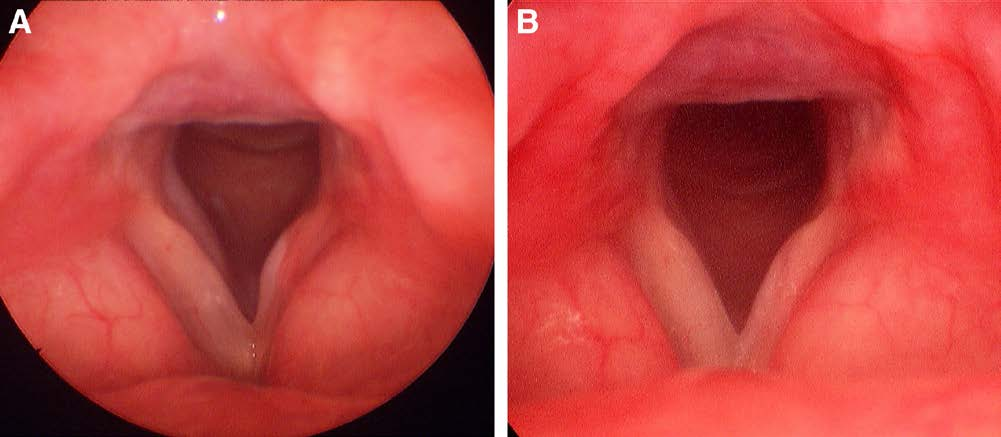
Postoperative laryngoscopic views at follow-up. (A) Laryngoscopic image obtained 2 months after surgery, showing well-healed glottic mucosa without evidence of recurrence. (B) One-year postoperative image demonstrating no evidence of granuloma or residual lesion.

With the patient in the sniffing position, the VL blade was inserted and positioned in the vallecula. Remifentanil was infused at a target effect-site concentration (Ce) of 2 ng/mL. The patient exhibited neither a gag reflex nor moderate-to-severe coughing. VL confirmed the presence of sufficient space to the right of the mobile granuloma consistent with the preinduction FOB findings (Fig. 2C–D), which allowed the passage of a 5.5 mm ID ETT. During deep exhalation, the mass retracted from the glottis, enabling successful advancement of the tube into the trachea. After successful intubation, general anesthesia was immediately induced by intravenous administration of propofol at a target Ce of 4 μg/mL, followed by 40 mg of rocuronium. General anesthesia was then maintained while adjusting the Ce of propofol and remifentanil according to the patient’s intraoperative BIS and vital signs, respectively. The surgeon successfully excised the mass during the laryngeal microscopic surgery (Fig. 3A–C), and the total surgery was completed in approximately 10 minutes. At the outpatient visit 2 weeks after surgery, the patient reported a slight cough and a tickling sensation in the throat but had no dysphonia or dyspnea. Postoperative laryngoscopy revealed a clear surgical field at 2 months and 1 year postoperatively (Fig. 4A–B). As the clinical data were deidentified, the case was granted an exemption from ethical approval by the Institutional Review Board of Kyungpook National University Chilgok Hospital in Daegu, Republic of Korea (KNUCH 2025-07-002). The patient provided informed consent for the publication of this case report.

## 3. Discussion

Granulomas are benign lesions that can develop as part of the wound healing process. In the pharynx or larynx, they are often associated with mechanical trauma such as endotracheal intubation, voice strain, and inflammatory stimuli, including gastroesophageal reflux, infection, and postnasal drip.^[1,2]^ However, vocal process granulomas, which commonly arise at the posterior one-third of vocal fold,^[1]^ are typically multifactorial, making classification by etiology difficult, except in cases clearly linked to intubation. Initial treatment involves conservative management, such as voice therapy, dietary modification, anti-reflux medication, antibiotics, and steroid injections, based on the underlying cause.^[1]^ Surgical intervention is generally reserved for cases where conservative therapy fails after several months, when airway obstruction results in respiratory distress, or when histological evaluation is necessary. In our patient, hoarseness was the initial presenting symptom, and a small granuloma was noted during the first otolaryngology clinic visit 2 months prior. At that time, observation was selected. However, the lesion became progressively enlarged, eventually leading to critical airway obstruction with accompanying dyspnea, necessitating emergency surgery.

Preoperative laryngoscopy revealed a large granuloma nearly occluding the glottic inlet. Despite the severity of the obstruction, limited airflow was possible as the lesions shifted slightly outward from the glottis during exhalation. In this context, inducing general anesthesia would have eliminated spontaneous respiration, necessitating positive pressure ventilation. Muscle relaxation would have further abolished vocal cord movement, increasing the risk of complete obstruction, with positive pressure potentially forcing the lesion into the glottis. Moreover, if a narrow gap allowed air entry during inspiration but prevented its release during expiration, a ball-valve type obstruction could develop, leading to air trapping and subsequent tension physiology.^[4]^ Therefore, preserving spontaneous ventilation was crucial until a secure airway could be established, necessitating awake airway management prior to induction. Typically, awake tracheostomy performed by an otolaryngologist is considered the safest option. However, in this case, the surgeon was concerned about tracheostomy-related complications and requested an initial attempt at tracheal intubation by the anesthesia team.

In some cases with anticipated difficult airways, such as those involving morbid obesity,^[5,6]^ imaging in different positions, including the lateral decubitus position, has been reported to aid in assessing the effect of gravity on airway patency. Positional changes have also been reported to facilitate tracheal intubation.^[7,8]^ However, in the present case, the patient presented with dyspnea, and only an essential flexible laryngoscopic examination was performed; preoperative computed tomography imaging was omitted. As shown in Figures 1B and 2, the granuloma was large, occupied most of the glottic inlet, and moved dynamically in and out of the glottis during respiration. Considering the lesion’s location, size, and mobility, lateral positioning was unlikely to produce a meaningful improvement in airway patency. Therefore, airway management focused on awake intubation with continuous visualization rather than positional modification.

In cases of an anticipated difficult airway, awake FOB-guided intubation has traditionally been considered the first-line approach in anesthesiology.^[9]^ FOB is advantageous in situations involving limited mouth opening or when there is a need for nasal intubation,^[10]^ and it allows the operator to flexibly navigate around anatomical structures. Moreover, when an external monitor is used, the view can be shared among multiple operators, facilitating team coordination. However, in cases such as ours, the structural characteristics of the FOB may limit its utility. Because the ETT is mounted over the bronchoscope shaft, it remains outside the camera’s field of view, making it difficult to visualize the lesion, glottis, and tube simultaneously during advancement. In addition, the bronchoscope’s extreme flexibility provides insufficient mechanical support, increasing the risk of tube deviation or failure to pass through the glottis when maneuvering around a lesion.

In contrast, VL offers several advantages. Through gentle traction, the blade can displace surrounding anatomical structures and create a stable pathway,^[3]^ allowing a styletted ETT to be advanced in a controlled manner once the glottis is visualized. The camera at the blade tip provides a wider field of view, enabling real-time assessment of the lesion’s position and movement, as well as simultaneous visualization of the lesion, glottis, and tube,^[3]^ which is particularly beneficial when managing a large, mobile lesion. In addition, awake VL-guided intubation allows flexible selection of tracheal tube size under direct vision,^[3]^ which is especially useful when a smaller tube is required to navigate a narrowed glottic opening caused by the lesion. In our case, the giant pedunculated granuloma prolapsed into the glottic inlet during respiration, and VL enabled continuous dynamic monitoring to time tube insertion precisely during exhalation, when the lesion shifted outward. Accordingly, VL-guided awake intubation was selected as the technique of choice.

Previous reports have demonstrated that direct laryngoscopy can also be successful,^[10,11]^ but VL provides superior visualization through its distal camera^[12]^ and may reduce the risk of lesion injury or dislodgement. Furthermore, the shared view facilitated real-time collaboration between anesthesiologists and otolaryngologists, allowing immediate joint decision-making if difficulties arose.^[12]^ Several case reports have emphasized avoiding manipulation or forceful displacement of the lesion into the airway, as such maneuvers may increase the risk of bleeding, trauma, or airway compromise.^[3,13–15]^ Consistent with these reports, we minimized lesion manipulation and used VL to continuously monitor lesion movement in real time, enabling safe and precise intubation.

Awake intubation requires effective topical or needle-based local anesthesia, depending on the targeted reflexes and anatomical regions.^[10]^ To suppress the gag reflex, topical anesthesia of the posterior tongue and soft palate, as well as glossopharyngeal nerve blocks, may be considered. To suppress the cough reflex and prevent laryngospasm, superior laryngeal nerve blocks targeting the epiglottis and supraglottic larynx are useful. Transtracheal blocks are effective for anesthesia of the subglottic region and trachea. In this case, in addition to our standard protocol – using oropharyngeal lidocaine spray and a transtracheal block – we also performed a superior laryngeal nerve block to minimize cough and discomfort from VL blade contact during intubation.^[10,11]^ To enhance patient comfort and blunt the stress response, remifentanil infusion was administered for analgesia. Recognizing the potential for unexpected sedation, respiratory depression, or apnea, we preemptively applied high-flow nasal cannula (HFNC) to facilitate apneic oxygenation, a method that has recently gained recognition as a valuable adjunct in managing complex airways.^[16,17]^

This case exemplifies the critical need for awake airway management in patients with vocal process granulomas that have progressed to near-complete airway obstruction. When a large, mobile granuloma intermittently occludes the glottic inlet during spontaneous respiration, conventional induction of general anesthesia poses a significant risk of total airway collapse and the development of tension physiology. In such cases, maintaining spontaneous ventilation until the airway is secured is essential. Compared to FOB, VL provides superior visualization of the lesion’s position and movement, offering a safer approach to intubation while minimizing trauma or dislodgement. Successful awake intubation requires comprehensive preparation, including topical and nerve block-based local anesthesia, remifentanil-based analgesia to minimize discomfort and airway reflexes, and the use of HFNC for apneic oxygenation. Importantly, these strategies should be executed within the framework of close multidisciplinary collaboration between anesthesiology and otolaryngology departments to ensure patient safety and optimize outcomes.

## 4. Conclusion

This case highlights the importance of individualized airway management in patients with vocal process granulomas causing critical airway obstruction. In cases where a mobile lesion intermittently occludes the glottis, conventional induction of general anesthesia may precipitate total airway collapse and cause life-threatening complications. Awake intubation techniques, particularly those performed under VL guidance, offer a safer alternative by allowing real-time visualization of the dynamic airway anatomy while preserving spontaneous ventilation. Successful airway management in such cases requires meticulous preparation, including targeted local anesthesia, use of analgesic agents such as remifentanil, and apneic oxygenation via HFNC. Furthermore, close interdisciplinary coordination between anesthesiology and otolaryngology teams is essential to ensure a prompt and safe response to potential airway failure. This case reinforces the value of awake VL-guided intubation as a viable and effective strategy in managing anticipated difficult airways due to obstructive laryngeal pathology.

## Author contributions

**Conceptualization:** Kyung-Hwa Kwak, Sung-Hye Byun.

**Data curation:** Jinyoung Oh, Sou-Hyun Lee, Sung-Hye Byun.

**Investigation:** Sou-Hyun Lee, Jongone Park.

**Resources:** Jinyoung Oh, Jongone Park.

**Supervision:** Kyung-Hwa Kwak, Younghoon Jeon.

**Visualization:** Sung-Hye Byun.

**Writing – original draft:** Jinyoung Oh, Sung-Hye Byun.

**Writing – review & editing:** Sung-Hye Byun.
